# Diseases of the Scalp.—The Syphilides

**Published:** 1885-02

**Authors:** A. R. Davidson

**Affiliations:** Professor of Medical Chemistry and Dermatology, Niagara University


### Diseases of the Scalp.—The Syphilides.

### A CLINICAL LECTURE DELIVERED AT THE HOSPITAL OF THE SISTERS OF CHARITY.

By A. R. Davidson, M. D.,
Professor of Medical Chemistry and Dermatology, Niagara University.

Gentlemen—This man presents himself with a disease of the scalp. It will be an aid to you in your diagnosis of these cases, to remember that the principal cutaneous diseases, which affect the scalp, can almost be counted upon the fingers of one hand. We may have eczema, psoriasis, seborrhœa, pityriasis, tinea tonsurans, tinea favosa.

The last two are due to the presence of a parasite which thrives upon the superficial or epithelial elements of the skin. Tinea tonsurans, tinea circinata and sycosis, though presenting somewhat different features, are all due to one and the same cause, the trichophyton, a vegetable parasite belonging to the fungi, and differing from the algæ as being devoid of chlorophyl and being unable to assimilate inorganic matter. Tinea favosa is also due to the development of a vegetable fungus, the achorion schoenleinii; it is characterized by the formation of "favus cups." These are peculiar, circumscribed, dry, yellowish umbilicated masses, which at first are firmly attached to the surface of the skin, but later may be easily detached. The microscope shows them to be made up of mycelium and spores, also epidermal cells and debris. It is, in this country, one of the rarer diseases, and I will probably not have an opportunity of showing it to you, but in *lieu* of that, this plate will give you an excellent idea of its appearance.

You are sufficiently familiar with the appearance of tinea tonsurans to know that this case presents no resemblance to that disease.

*Pityriasis* is a chronic squamous disease of the skin in which the scales are branny and are seated on a non-infiltrated surface.

The ordinary condition known as pityriasis capitis is due to an increased secretion of the sebaceous glands, and is, in no sense of the word, a pityriasis.

Later on, however, as a consequence of long continued catarrh of the sebaceous glands, there may be a secondary involvement of the epidermis. The scales are, then, true squamæ, dry, corneous and epithelial, but even this condition is better designated by the term alopecia furfuracea, as it is one of the commonest causes of permanent baldness.

*Seborrhœa* is a condition due to increased secretion of the sebaceous glands, and also, in most cases, to a change in the quality of the sebum poured out. As the sebaceous glands are mainly appendages to the hair, seborrhœa capilitii is not uncommon, and is vulgarly known as dandruff. As it never presents features in common with the present case, we will pass it over without discussion at present.

We have now only eczema and psoriasis to consider. I have shown you cases of both these diseases, and you know that the recognition of a pronounced case of either eczema or psoriasis is made with ease, even by those unskilled in cutaneous diseases; but in the atypical forms you may often have occasion for doubt. Without entering, at this time, into the general characteristics of these two diseases, a consideration of which will usually clear up any obscurity which might arise from the eruption itself, I will agree with those of you who have already expressed an opinion that this case most resembles a psoriasis. But let me call your attention to some important points: First, you will notice that the scales are dirty whitish and not silvery and lustrous in color; indeed, many of the lesions are more of the character of crusts, being agglutinated by pathological exudations from the patch. When I remove the scales, the subjacent skin is of a dull red color, not so bright a tint as is usually seen in psoriasis. You will notice, too, that there has been considerable loss of hair, and that what remains is dull and dry, and easily pulls out. Now, if this is a case of psoriasis, we are almost sure to find the eruptions upon other parts of the body, the sites of preference of the disease being over the extensor surface of the extremities, and especially about the elbows and knees, where it is decidedly most common. The man will now remove his shirt, and we will see what further knowledge we can gain by an inspection of his body. At once you see that there is no indication of psoriasis, but, instead, it is pretty uniformly spotted with a macular eruption, not elevated above the general level of the integument, and of a dull, mulberry hue.

The maculæ, you will notice, are mostly round. The color does not disappear under pressure with the finger, and he says the eruption causes no itching or other subjective sensations.

This man may now retire. I have allowed this man to withdraw because he was unwilling to be made the subject of remark upon the effects of a disease which is not usually looked upon with much pride by its possessor. You doubtless have all recognized this as a case of syphilis, but just here let me say that you must not charge every individual presenting symptoms, whatever his or her personal antecedents or character, with having indulged in illicit sexual intercourse. Up to a comparatively recent period it was believed that the chancre was the only contagious lesion. We now know that the generalized lesions, especially those which are suppurative in character, are among the frequent sources of contagion. Thus, infection may occur from the passage of a cigar or pipe from mouth to mouth, from the use of common utensils, from sleeping in the same bed; surgical instruments may convey contagion, or vaccination. Mucous papules and patches are frequent sources of contagion, and next to them the pustular syphilodermata, but do not forget that whether the infection be derived from a chancre or a secondary lesion, the initial manifestation of syphilis is always a chancre.

This man presented himself to me six months ago with all the secondary symptoms beautifully developed. Under specific treatment the rash disappeared and he was soon in apparent health. He was particularly ordered to continue the treatment, but considering himself well he stopped all medication. A week ago I was called to see him and found him in much the same condition he is now except that he then had an iritis which has now disappeared. For this he had consulted an ophthalmologist, who had again put him on a specific treatment. The case is an interesting one to you, as showing the recurrence of the original eruption, and also an instance of the manifestations of a disease which are protean in character. On the body we have the macular, on the scalp a well-marked squamous syphilide resembling psoriasis and often called syphilitic psoriasis. Syphilis has been described as an imitator of other diseases; in its manifestations upon the skin it may present the different lesions common to other cutaneous affections, but there are always certain differences for a study of which we must be familiar with the syphilitic mode of disease.

Syphilis is a slow-staged specific fever, the result of a particulate poison which is conveyed only by contact. It runs a course tolerably definite in its main phenomena, but varying in character and detail. It is divided into various stages. Inoculation of the virus is followed by an interval varying from ten days to a month, in which no signs are manifested of its presence. This is the period of incubation. Well authenticated cases prove that the period may be extended to forty-five days, but we believe that the development of the chancre never occurs in a shorter period than ten days after infection; occasionally a slight redness indicates some local irritation at the point where the poison was introduced, which, however, speedily subsides, and unless chancroidal virus has been mixed with that of syphilis, nothing, as a rule, is seen; at the end of this period, which, you will remember, is about four weeks, the local sore, a chancre, has usually become characteristic. Then, unlike the other exanthemata, there follows a second stage of incubation which terminates in a month or six weeks with the appearance of a rash and fever.

The first stage is called the primary, and has for its phenomena the chancre and the bubo; its usual duration, including the stage of local incubation, is about two months. You will have abundant opportunities to study these initial manifestations of the disease, and time will not allow me to speak of the characteristics of the true or false sore. The bubo, associated with the true chancre, is usually like it, very hard, and shows little tendency to inflame or suppurate. That which attends the false sore not unfrequently suppurates; no constitutional symptoms follow the chancroid. It is often extremely difficult to discriminate between the chancre and chancroid, and in doubtful cases no opinion should be ventured until the incubation stage (one month) is at an end.

The secondary stage commences with a slight fever, followed by an eruption on the skin, sores in the throat and mouth, aches in bones, joints and muscles, and occasionally inflammation of the eye. All parts of the body are liable to be affected, but all the inflammations produced are usually transitory and often very slight.

The eruptions on the skin in the secondary stage are very various in character. Roseola is often the earliest, and most commonly followed by the papular and squamous syphilides, which are well illustrated in these plates, but we may have eruptions resembling psoriasis, lichen, impetigo, acne, rupia, and many others; ulcers in the tonsils, sores on the cheeks or on the tongue and lips, and condylomata at the anus are also common. During the time the eruptions are making their appearance, chronic enlargement, with induration of the lymphatic glands throughout the body, are not uncommon, as, also, slight periosteal swellings, but they do not constitute large nodes, as in the tertiary stage. The patient is usually anæmic and thin, but not necessarily so.

All secondary symptoms are, as a rule, symmetrical. The course of syphilis is so much modified by specific treatment that it is difficult to assign correctly the duration of the secondary stage; it probably varies from a month or two, to a year or more. When it has come to an end the patient may never suffer again from any symptoms of the disease, or he may pass on directly to the development of those symptoms which are classed as tertiary. During the interval which intervenes between the secondary and tertiary stage, occasional relapses of the secondary symptoms may occur. The so-called psoriasis palmaris and sores on the tongue are the commonest symptoms, but we also have occasional relapse of general eruptions, an instance of which we have had in the man before us.

In other cases the tertiary symptoms may appear before the secondary signs have subsided. Thus, there is no distinct line of demarkation between secondary and tertiary syphilis. The division into stages is arbitrary, but convenient; when the tendency to the formation of local, and usually unsymmetrical inflammation begins, we commonly say that the tertiary stage has set in. It lasts, unless cured, the rest of the life.

The most characteristic pathological product of this stage is the gumma, or, as it is sometimes called, the syphiloma or syphilitic granuloma. They affect the testes, the liver, the meninges of the brain, the cellular tissue, the muscles, the periosteum, the choroid or the skin. In the skin they produce serpiginous or lupoid affections, and in the periosteum nodes. Wherever they begin they are prone to be serpiginous, that is, to creep at their edges. When this action occurs in the central parts of the nervous system, it may produce such maladies as paraplegia, locomotor ataxia, ophthalmoplegia, and the like.

During the primary and secondary stages the disease is violently contagious. During the secondary it is transmissible to offspring, while during the tertiary it is probably neither contagious nor transmissible. Tertiary inflammations are usually not symmetrical, but they are locally self-infecting and may spread indefinitely, both as to extent and time, showing no tendency to spontaneous disappearance, as the primary and secondary ones do.

To further impress upon you the sequence of events in syphilis, I will give you Fournier's striking analysis, in which its apparition and development is likened to a "drama" with three acts:

*First Act*—Contamination. The virus penetrates the organism by one mode or another.

*First Interval*—Apparent repose of the organism—incubation. Nothing appreciable betrays the disease, as yet.

*Second Act*—Introduction at the point where the virus has penetrated, and only here, of a lesion called initial, which, for the time, constitutes the only expression of the disease.

*Second Interval*—Another period of repose of the organism. The initial lesion continues to be the only symptom by which the disease is expressed.

*Third Act*—Explosion of multiple and disseminated lesions, beyond and outside of the seat of contamination. This is the period of visible generalization of the disease.

From this description of the disease, which is a mere outline of its distinctive features, you will recognize that lesions of the skin may appear at any period in the course of syphilis, being among the earliest symptoms, and not unfrequently among the latest. They are, however, much more frequent during the first two years after infection. Syphilitic eruptions are caused by two distinct morbid processes, hyperæmia and cell infiltration. The hyperæmic or erythematous syphilides present several varieties and are peculiar to the earlier stages, being rarely seen later than two years after infection. They may resemble, in their lesions, most of the other diseases of the skin, as has been already said. It is the syphilitic behavior, rather than the syphilitic lesion, which guides us in our diagnosis. Some of the peculiar features of the early syphilides are—

*Palymorphism*—A patient may show maculæ, dry and moist papules, pustules, scales, crusts, etc., all associated together. It is quite different with non-syphilitic eruptions, which usually present a tolerably uniform character.

*The peculiar color of syphilitic eruptions*—The syphilitic tint is usually described as of a copper color, or as like the color of a slice of lean ham. This is well illustrated in this man, but you must not place too much dependence on this characteristic. There is no color peculiar to the syphilodermata which may not be seen in other diseases of the skin. The macular syphiliderms, which are the most frequent and earliest expressions of cutaneous syphilis, are at first usually of a pale rose color ; afterwards the color deepens, and as the eruption pigments and begins to pass away, the brownish color is developed ; at first the color disappears upon pressure, but later is persistent.

The ham color is usually most marked on papules, while the coppery tint is more apt to be observed in tubercles.

*The peculiar rounded or circinate form* which the lesions very commonly present is often a valuable aid to diagnosis. Again, *their course*, as compared with that of simple eruption, is marked by chronicity and absence of inflammatory features, and of itching and pain—important diagnostic features.

*Their amenability to the curative influence of mercury*—A questionable eruption, which yields to this touchstone, may, with certainty, be regarded as syphilitic.

But, gentlemen, I have detained you over the hour, and I will say but a word as to treatment. We will place this man under the influence of mercury. The choice of the form in which it shall be administered is often little more than a matter of personal preference. There is sometimes an advantage, during a prolonged mercurial course, in using different preparations at different periods, as one long continued seems to lose its effect.

I am myself partial to the so-called "tonic" treatment, brought into prominence by the writings of Keyes.

We will give this man a pill containing one-sixth of a grain of proto-iodide of mercury, after each meal, gradually increasing the dose, until, upon striking the jaws together, a little sensitiveness of the teeth is manifested. Having thus ascertained the patient's susceptibility to mercury, we will divide the full dose by half, and will recommend its continuance for six months to a year. When all symptoms have subsided, the quantity may be still further reduced, say one-third of a full dose, and this dose persisted in for at least six months after every indication of the disease has disappeared.

It is often difficult to induce patients to follow out so prolonged a course of treatment. The symptoms once fairly gone, the patient gets tired of the continuous dosing, and if he stops too soon you are likely to have him back on your hands for the treatment of tertiary symptoms.

